# Machine Learning‐Based PAT Tool for Real‐Time Residual Moisture Monitoring in Lyophilized Biopharmaceuticals Using NIR Spectroscopy

**DOI:** 10.1002/bit.70302

**Published:** 2026-07-12

**Authors:** Samuele Fiorenza, Mariapia D'Urso, Ambra Massei, Nunzia Falco, Davide Fissore, Enza Torino

**Affiliations:** ^1^ Department of Information Technology and Electrical Engineering University of Naples Federico II Naples Italy; ^2^ Centro di Ricerca Interdipartimentale sui Biomateriali, CRIB University of Naples Federico II Italy; ^3^ Department of Chemical, Materials and Production Engineering University of Naples Federico II Naples Italy; ^4^ Université Paris‐Saclay, CNRS, ENS Paris‐Saclay Laboratoire Méthodes Formelles Gif‐sur‐Yvette France; ^5^ Global Drug Product Development Merck Serono S.p.A. Rome Italy; ^6^ Dipartimento di Scienza Applicata e Tecnologia Politecnico di Torino Turin Italy

**Keywords:** lyophilized biopharmaceuticals, machine learning, near‐infrared spectroscopy, process analytical technology, residual moisture monitoring

## Abstract

There is a growing demand in the pharmaceutical industry to optimize analytical methodologies, reducing time and resource consumption while ensuring rapid process development and cost‐efficient manufacturing. Indeed, Food and Drug Administration (FDA) introduced the Process Analytical Technology (PAT) as an initiative enabling manufacturers to measure and control in real‐time processes based on the Critical Quality Attributes (CQAs) of the product. This study explores the implementation of PAT as a strategic approach in the lyophilization process design and optimization. In this sense, Residual Moisture (RM) was selected as a CQA to evaluate lyophilized biopharmaceutical products, due to its impact on stability. Traditionally, RM quantification is obtained by Karl‐Fischer (KF) titration, a destructive technique. Recently, Near‐Infrared Spectroscopy (NIRS) was proposed as a non‐destructive alternative; however, its effectiveness is hindered by spectral complexity and non‐linear moisture‐spectra relationships. Here, PAT tool integrating NIRS with Machine Learning (ML) is developed to enable rapid, at‐line RM assessment, handling the complexity of non‐linear relationships between RM values and spectral features. Different ML models – Partial Least Squares (PLS), Support Vector Regression (SVR), XGBoost, and an ensemble PLS‐SVR approach – were trained on placebo samples and validated on drug products. SVR outperformed PLS at lower moisture levels, while PLS was more accurate at higher RM ranges. The ensemble model combining both approaches improved predictive accuracy across the full RM range tested. External validation using literature‐sourced datasets, including placebo and simulated drug products with different excipients, confirmed the model's robustness and adaptability. Furthermore, a minimal dataset augmentation strategy enhanced model specialization for specific formulations. The proposed NIRS‐ML ensemble model offers a scalable, non‐destructive solution for real‐time RM monitoring, supporting pharmaceutical manufacturing within the PAT framework. Its adaptability across formulations highlights its potential as a versatile tool for lyophilized biopharmaceutical manufacturing pipelines.

## Graphical abstract

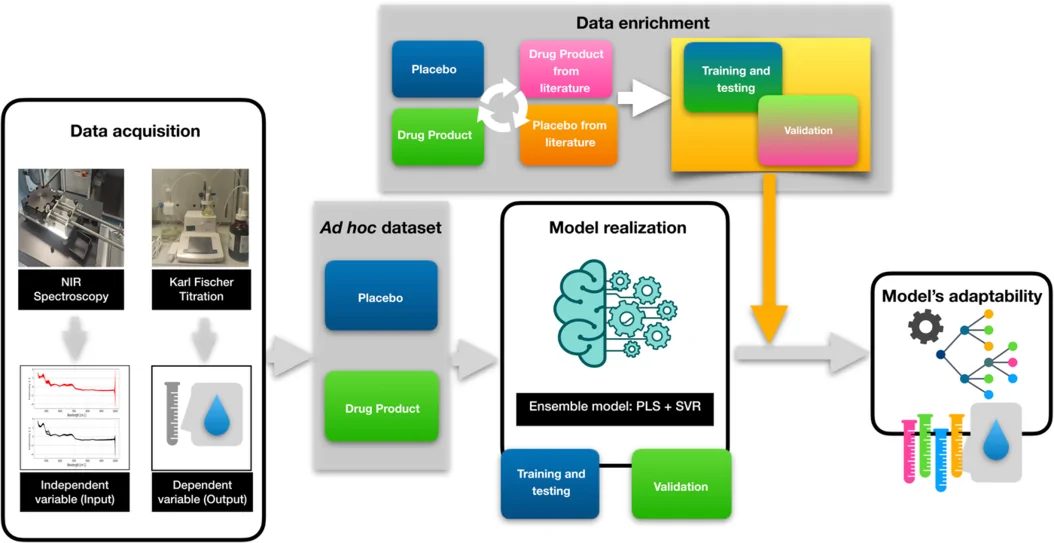

## 1 Introduction

The pharmaceutical field is investing tremendous efforts in optimizing the development of new Drug Products (DPs), a process characterized by high complexity and substantial investment. Each phase – from early research to clinical trials, regulatory approval, and large‐scale manufacturing – has a great impact on the final product quality. This process is time‐consuming and costly, spanning 15 years on average and costing $1–2 billion (Hughes et al. 2011; Sun et al. 2022).

Despite these efforts, the failure rate of drug candidates remains considerably high, with nine out of ten failing during drug discovery and phase I, II, or III clinical trials (Sun et al. 2022). A primary challenge within drug development is ensuring the drug stability, defined as the ability of a product to maintain its original properties from manufacture through its expiry date (Wong and Datla 2005). This concept is extended throughout the period of development, production, storage, and use of the drug, thus resulting in the general classification within pre‐market stability and commercial stability (Wong and Datla 2005).

Biopharmaceutical development is particularly costly and time‐expensive, especially when employing the traditional and resource‐intensive approaches required for process development, process manufacturing and stability studies (Wang and Ohtake 2019). Standard methods, indeed, do not allow real‐time process monitoring and adjustments during manufacturing, potentially resulting in batch failures. This has led to a demand for in‐line or at‐line analytical tools capable of real‐time monitoring, as emphasized by the FDA's Guidance for Industry on Process Analytical Technology (PAT) (Pisano and Fissore 2023; U.S. Department of Health and Human Services, Food and Drug Administration 2004, CDER, CVM, and ORA, “Guidance for industry PAT ‐ A framework for Innovative Pharmaceutical Development, Manufacturing and Quality Assurance,” ed.). The abovementioned PAT framework encourages the development and adoption of innovative tools for continuous process monitoring and control, to enhance product quality assurance and reduce manufacturing setbacks (U.S. Department of Health and Human Services, Food and Drug Administration 2004, CDER, CVM, and ORA, “Guidance for industry PAT ‐ A framework for Innovative Pharmaceutical Development, Manufacturing and Quality Assurance,” ed.). The applications of the PAT approach are rapidly expanding across diverse domains, from innovative immunotherapy (Thite et al. 2025) to the emerging gene therapy (Williams et al. 2023), while also transforming the modelling of traditional processes (Barhate 2024) and driving advances in industrial machine development (Kim and Han 2025) Recent studies have explored the use of advanced PAT tools, such as on‐line ultra‐performance liquid chromatography and multimode fluorescence spectroscopy, for characterizing biopharmaceutical critical quality attributes (CQAs) in real‐time, including total protein concentration and ratio of individual monoclonal antibody (mAb), demonstrating their ability to streamline analytical workflows and reduce turnaround time (Godbole et al. 2025).

Approximately 50% of the FDA‐approved drugs have been prepared by lyophilization (Pardeshi 2023; Chen and Wang 2007), including three out of the ten highest selling DPs worldwide in 2016 (Izutsu 2018). Lyophilization, commonly known as freeze‐drying, is a fundamental process in the pharmaceutical setting to enhance the stability and the shelf‐life of heat‐sensitive compounds such as vaccines, proteins, and other biologically active substances (Izutsu 2018; Tang and Pikal 2004; Vass et al. 2019). Plus, freeze‐drying process also ameliorates the handling and distribution of pharmaceutical products (Pikal 1994; Carpenter and Chang 2020). Removing water from the product at low temperatures (e.g., −20°C) and under vacuum conditions ensures that the pharmaceutical product remains stable over extended periods (O'Sullivan et al. 2022). Indeed, the success of the lyophilization process is dependent on the residual moisture (RM) content in the final product, which is one of the most impacting CQAs (Pardeshi 2023; Miller et al. 2018; Hageman 1988). Even a small quantity of the latter above the target value can negatively impact the stability, potency, and the overall quality profile of pharmaceuticals (Pardeshi 2023; Miller et al. 2018; Jameel et al. 2021). Therefore, accurate measurement of RM is pivotal in ensuring product safety and compliance with regulatory standards (EMA n.d. “ICH Q8 (R2) Pharmaceutical Development ‐ Scientific Guideline.”). While Karl‐Fischer (KF) titration emerged to be the standard analytical method for RM quantification, it is destructive, time‐ and reagents‐consuming, requires significant manual input, and cannot be implemented at‐line thus highlighting the need for non‐invasive alternatives (Bruttel and Schlink 2003).

To provide an overview of the available techniques for RM determination in lyophilized samples and their applicability to PAT, Table 1 summarizes the main offline and at‐/in‐line methods, together with their advantages and limitations.

**Table 1:** Available techniques for RM determination in lyophilized samples.

Method	Typical mode	Destructiveness	Advantages	Limitations	Refs.
KF titration	Off‐line	Yes	Reference method; high sensitivity/accuracy	Destructive; time/reagent consuming; not suitable for at‐/in‐line PAT; limited sampling per batch	Theimer and Pavelek (1968)
Loss on drying	Off‐line	Yes	Simple, low cost	Non‐specific; risk of thermal degradation; limited comparability across formulations	Savage et al. (1998); Razvi et al. (2021)
Thermogravimetric analysis	Off‐line	Yes	Can separate mass‐loss events; useful for formulation studies	Typically offline/slow; not strictly water‐specific; not routine PAT	May et al. (1982); Ottoboni et al. (1998)
NIR spectroscopy	At‐line/potentially in‐line	No	Rapid, non‐destructive; minimal to no sample prep; strong sensitivity to water; mature PAT adoption	May require calibration/chemometrics; sensitive to matrix/physical effects	Lin and Hsu (2002); Massei et al. (2023)
Mid‐IR/FTIR spectroscopy	Off‐line/at‐line	Yes/No	High chemical specificity; strong water absorption	Through‐vial implementation is challenging; ATR is contact based; less practical for routine non‐invasive PAT in vials	Affleck et al. (2021)
Raman spectroscopy	At‐line	No	Orthogonal to NIR; strong for solid‐state and formulation characterization	Water Raman signal is relatively weak; fluorescence can interfere; quantification of low RM can be challenging; longer acquisition/complexity	De Beer et al. (2009)
Terahertz spectroscopy	At‐line	No	Very sensitive to water; can probe cake structure/porosity	Less established in pharma manufacturing; higher cost/complexity; method standardization still evolving.	Huang et al. (2023)

Among those analytical strategies, Near‐Infrared Spectroscopy (NIRS) has been tested as a promising alternative to KF titration for the determination of RM in lyophilized pharmaceuticals (Lin and Hsu 2002; Grohganz et al. 2011; Jamrógiewicz 2012). Specifically, NIR spectroscopy (NIRS) is a widely used analytical technique in the pharmaceutical industry due to its ability to provide rapid, non‐destructive, and real‐time analysis of various CQAs, including moisture content (Jamrógiewicz 2012; Igne and Ciurczak 2021). It leverages the interaction of the NIR light (780 nm to 2500 nm) with molecules, where molecular overtones and combinations of fundamental vibrations (*e.g*., O‐H, N‐H, and C‐H bonds) occur, particularly those characteristics of the water molecules (Burns and Ciurczak 2007). The absorption of NIR light by these molecular bonds leads to the generation of the spectrum, that can be used to infer the moisture content of a sample. However, direct quantification of RM using NIR spectroscopy can be challenging due to the complexity of the spectra, which is often exhibiting absorption bands overlapping, and because of the non‐linear relationships between the spectral features and the moisture levels.

To address these challenges, data analysis techniques, such as machine learning (ML), have been increasingly employed to model the complex relationships between NIR spectral data and moisture content (Lakeh et al. 2020). By developing robust and accurate predictive models, ML can effectively address the inherent complexity and non‐linearity of NIR data. Machine learning includes a range of algorithms and statistical models designed to perform specific tasks by learning from data (Sarker 2021). One of its key strengths lies in the ability to automate data analysis once the model has been trained, making it highly suitable for predictive analytics, data mining, and image processing (Manesh 2019). In pharmaceutical research, ML has demonstrated significant potential in enhancing prediction accuracy, optimizing manufacturing processes, and improving Quality Control (QC). For instance, Bobba et al. proposed the use of Fourier‐Transform NIRS as a fast and non‐destructive technique for off‐line statistical QC application in freeze‐drying processes (Bobba et al. 2021). ML models have shown exceptional capabilities at analyzing high‐dimensional datasets, such as NIR spectra, and have been successfully applied to predict various pharmaceutical properties, including the RM in lyophilized products. Several studies have highlighted the promise of NIRS combined with ML for moisture prediction in lyophilized products. Particularly, Massei and co‐workers suggested the possibility of obtaining moisture predictions in freeze‐dried pharmaceuticals by using an Artificial Neural Network (ANN) based model coupled with NIR spectroscopy (Massei et al. 2023). Nevertheless, there is still the need for developing more robust strategies, especially at low RM values (below 1%) that are applicable in an industrial context, where greater precision is required to meet the strict QC standards required in pharmaceutical manufacturing. Indeed, such a low margin of error is critical to guarantee the reliability of moisture content predictions, as even slight deviations could significantly impact the stability and efficacy of lyophilized products (Miller et al. 2018; Jameel et al. 2021).

This study lies within the PAT framework, aiming to optimize analytical workflows for residual moisture prediction in lyophilized biopharmaceuticals. By leveraging machine learning models to correlate NIR spectral data with residual moisture levels measured through Karl‐Fisher titration, this approach provides a rapid, non‐destructive solution for monitoring RM and improving process control. Specifically, ML models employed are Support Vector Regression (SVR), Partial Least Squares (PLS), XGBoost, and an ensemble model consisting of a combination of SVR and PLS depending on the range of RM to be predicted. SVR and PLS were selected for the development of the ensemble method based on their performance and explainability, which is particularly valuable for pharmaceutical PAT applications where FDA is increasingly emphasizing the needs for explainable models (in compliance with ICH Q8 (EMA n.d. “ICH Q8 (R2) Pharmaceutical Development ‐ Scientific Guideline.”), ICH Q13 (ICH 2023a), and ICH Q14 (ICH 2023b)). The applicability of this approach is further demonstrated by predicting moisture levels in samples containing an Active Pharmaceutical Ingredient (API) without including these samples in the model training phase. Additionally, the study aims to showcase the generalization of the developed models by applying them to data with different NIR wavelength ranges, obtained from a previous study (Massei et al. 2023). Finally, this work demonstrates how the model can be fine‐tuned for a specific application by enriching the training dataset with a minimal number of experimental points closer to the problem at hand – for instance, samples containing a specific molecule or a particular combination of stabilizing excipients. This study contributes to the acceleration of the biopharmaceuticals transition from research and development stages to industrial production and clinics.

## 2 Materials and Methods

### 2.1 Samples Preparation

In this work, two different sets of samples namely Product 1 and Product 2 were employed. As listed in Table 2, Product 1 has no Active Pharmaceutical Ingredient (API) in the formulation, and it is also referred to as placebo. Product 2 presents the same excipients as Product 1 plus an amount of API, referred as Protein 1, in the order of micrograms/mL.

**Table 2:** Ingredients of Product 1 and Product 2.

Ingredient	Product 1 – quantity [mg/mL]	Product 2 – quantity [mg/mL]
$Na_{2}\mathrm{HP}O_{4}\cdot 2H_{2}O$	2.22	2.22
$Na_{2}H_{2}PO_{4}\cdot H_{2}O$	0.90	0.90
Sucrose	60	60
API	0	100 [mcg/mL]

All the experimental trials were performed in the laboratories of Guidonia Montecelio site of Merck Serono S.p.A. Rome – Italy (an affiliate of Merck KGaA, Darmstadt, Germany). Product 1 was prepared by dissolving 2.22 g of Disodium hydrogen phosphate dihydrate (supplied by Merck Millipore, code 106580), 0.90 g of Sodium dihydrogen phosphate monohydrate (provided by Merck Millipore, code 106349) and 60 g of Sucrose (provided by Merck Millipore, code 107651) in 1 L Milli‐Q Water. Each ingredient was weighed using the analytic balance XPR205 supplied by Mettler Toledo, previously calibrated.

Product 1 samples were then freeze‐dried by using a pilot freeze‐drier machine supplied by IMA Life, Italy. Different filling volumes were selected to ensure high‐quality spectral detection since it is important that the cake covers the entire detection area of the NIR probe to reduce detection noise. 100 vials were filled with 0.5 mL of placebo solution and the remaining 100 were filled with 1 mL of the same solution. Subsequently, they were arranged within the batch in a hexagonal pattern and pre‐stoppered. They were not completely sealed since, during lyophilization, the sublimated water had to exit from the vials to allow the decrease of free water content. Further details on the lyophilization process parameters are included in Supporting Information (Section S1). At the end of the process, the vials were crimped using a crimping machine to prevent re‐humidification.

Product 2 samples were provided by Merck Serono S.A. Rome – Italy (an affiliate of Merck KGaA, Darmstadt, Germany) already lyophilized in 2017. Then, 18 samples were stored at 5 ± 3°C for 6 years while the remaining 10 samples were stored at 25 ± 2°C for 6 years. An evaluation of the appearance of freeze‐dried cakes was conducted before use.

### 2.2 Karl‐Fischer Titration

Residual moisture values were quantified through the Karl Fischer titration, by using the C30S Coulometric KF Titrator supplied by Mettler Toledo. Prior to the measurements, the instrument was calibrated using a water standard. The value of the total RM was calculated by subtracting the value of humidity of the blank solution from the value of humidity registered in the reconstituted samples plus the analyte solution. The same procedure was repeated for each sample of Product 1 and Product 2 sets. The vials were weighed before and after reconstitution, which was done by injecting the optimal volume of reconstituting solution, by following internal Merck protocols. These data allowed us to evaluate the amount of water in terms of mg/vials and finally the residual moisture values. The equations used are the following (Equations (1), (2), (3), (4)):

(1) $$w_{\mathrm{rec}}=w_{b_{\mathrm{full}}}-w_{b}$$

(2) $$\rho_{\mathrm{rec}}=\frac{w_{\mathrm{rec}}}{V_{\mathrm{rec}}}$$

(3) $$\mathrm{Humidity}\left[\frac{\mathrm{mg}}{\mathrm{vial}}\right]=\frac{\left(H_{\mathrm{rec}}-H_{\mathrm{sol}}\right)V_{\mathrm{rec}}}{V_{\mathrm{inj}}}$$

(4) $$\mathrm{Humidity}\left[\%\right]=\frac{\mathrm{Humidity}\left[\frac{\mathrm{mg}}{\mathrm{vial}}\right]}{\mathrm{Cakeweight}}100$$

where $w_{b_{\mathrm{full}}}$ is the weight of the reconstituted vial, $w_{b}$ is the weight of the un‐reconstituted vials, $\rho_{\mathrm{rec}}$ is the density of the reconstitution solvent injected in a vial, $V_{\mathrm{rec}}$ is the volume of solvent injected to reconstruct a vial, $H_{\mathrm{rec}}$ is the humidity of the reconstituted sample recorded, and $H_{\mathrm{sol}}$ is the humidity of the solvent for reconstitution. The cake weight was computed by subtracting the weight of an empty vial plus the cup from the total weight of lyophilized vials. Since the value did not significantly change in different vials, one value was considered enough representative for the whole batch and thus taken as a reference.

A humidification step was performed on Product 1 samples in order to explore a wider range of humidity, since the expected value at the end of lyophilization is lower than 1%. Before proceeding with this step, it was crucial to ascertain the average amount of residual moisture present to understand the precise quantity of water needed to properly humidify the vials. For this reason, 10 placebo samples were tested using Karl Fischer titration and the collected values are reported in Table 3.

**Table 3:** Evaluation of the residual moisture in 10 samples of Placebo.

H_2_O KF [μg/inj]	H_2_O [mg/vial]	Final RM [%]
653.50	0.95	0.43
562.80	0.77	0.35
530.90	0.70	0.32
607.10	1.03	0.47
599.00	1.00	0.45
871.40	1.56	0.71
855.20	1.53	0.70
938.50	1.69	0.77
902.60	1.63	0.74
933.10	1.68	0.76

The average percentage value of residual moisture was approximately 0.60%, with a range of residual moisture from 0.32% to 0.76%. This variability could be attributed to the position of the vial within the batch during lyophilization as samples from the middle of the same batch line exhibited higher humidity values due to non‐uniform heat exchange within the vials and the heating shelf (Massei and Fissore 2023; Thompson and Fissore 2019).

The humidification consisted in unsealing some of the lyophilized vials and pipetting a microliter‐scale amount of water into the cap of each one. Then, each vial was positioned upside down and kept at room temperature for different durations, depending on the amount of water inserted, not longer than 3 h due to the hygroscopic nature of this specific cake.

At the end of the humidification process of Product 1, the obtained dataset is composed as follows (Table 4).

**Table 4:** Residual moisture ranges of Product 1 (after humidification) and Product 2 samples.

Dataset	Humidification	Residual moisture (RM) range
Product 1: 80 vials	No	0.30%–0.80%
Product 1: 3 vials	Yes	1.10%–1.40%
Product 1: 16 vials	Yes	1.40%–1.80%
Product 1: 11 vials	Yes	1.80%–2.20%
Product 2: 28 vials	No	0.30%–1.25%

### 2.3 NIR Spectra Acquisition and Data Elaboration

NIR spectra were collected by using a Fourier‐Transform NIR spectrometer (Antaris MX FT‐NIR, Thermo Fischer Scientific, Waltham, USA), equipped with an InGaAs detector and a halogen NIR source. Although the instrument supports acquisition up to 10,400 cm^−1^, the modality of acquisition of the spectra was in diffuse reflectance within the NIR wavelength range 10,000–4,000 cm^−1^, consistent with prior work (Massei et al. 2023). The algorithm of acquisition was previously implemented on the software Thermo RESULT; specifically, each spectrum resulted from the average of 96 scans to improve the Signal‐to‐Noise Ratio (SNR) of the measurements. The resolution was fixed at 8 cm^−1^. The spectra were collected by holding each vial upright as described in previous work by Bobba et al (Bobba et al. 2021). The values of absorbance obtained from this process were collected in a spreadsheet, whose rows reported the ID number of each sample, and the columns contained the wavelengths, to make the further data elaboration easier.

### 2.4 Dataset Preparation and Algorithm Development Strategy

The objective of the experimental approach was to obtain a dataset to be used in the machine learning algorithms development, to model the relations between the variations in the NIR spectra and the changes in the measured values of residual moisture.

During the algorithm development and validation, two distinct datasets were adopted: the first (referred to as *ad hoc* dataset) was specifically created for this study while the second was sourced from literature (Massei et al. 2023).

The *ad hoc* spectra collected were included in the wavelength range 10.000–4000 cm^−1^, but only a limited part of the data was involved in the algorithm dataset. As for the phase of Product 1‐based training and testing and Product 2 – based validation, the wavelength range 4000–4377 cm^−1^ was excluded from the datasets. This choice is attributable to the high amount of noise affecting the spectra, ultimately resulting in model performance penalization.

Regarding the development steps involving the dataset taken from literature (Massei et al. 2023), the wavelength range considered is 7100–4254 cm^−1^. In this case, the *ad hoc* spectra wavelength interval was restricted to the one of the external dataset (Massei et al. 2023), to ensure size compatibility and, thus, results comparability.

In order to increase the usability of the raw spectra dataset for predictive purposes, an additional step of data processing was needed. It consisted of a series of two techniques: a second‐order Savitzky‐Golay filter with a window size of 31 and a Standard Normal Variate (SNV). The first was applied to reduce data noise by preserving local features whilst the second performed numerical data scaling. Pre‐processed spectra are reported in Supporting Information.

The development of a Machine Learning algorithm is generally divided into two phases: training (or calibration) phase and testing phase. The two different categories of samples were specifically employed at different stages in the development of the model following the approach outlined in Table 5.

**Table 5:** Aim of the product used and number of samples.

Product	Number of samples	Aim
Product 1	129	Training
Product 2	28	External validation

From an industrial perspective, using placebo samples instead of drug products offers economic advantages and increased availability, allowing for obtaining a larger number of samples with lower efforts and costs. This is the reason why Product 1 samples were massively used for calibration purposes, which is the most resource‐intensive step. Conversely, it is important to highlight that this work was focused on developing a Machine Learning algorithm capable of assessing the Residual Moisture content in drug products. It is natural to consider that the presence of APIs in the training samples could introduce new features into the NIR spectra, exploitable by the algorithm to make more accurate predictions. In other words, the absence of drug products in the calibration set could represent a missed chance to enhance algorithm's performance. An external validation step was then performed. This final phase aimed to assess if the algorithm trained exclusively on placebo samples is capable of making predictions with sufficient accuracy by taking input spectra belonging to different kinds of samples with respect to the training one.

The whole set of samples is summarized in Table 6 below.

**Table 6:** Description of the whole sets of samples used.

Dataset name	Composition [% w/w]	Type of sample	Objective	Number of samples	Source
Product 1	Sucrose	Placebo	Training and testing	129	From current work
Product 2	6% Sucrose + < 0.01% Protein 1	Drug Product	Validation	28	From current work
SA05	6% Sucrose + 0.5% arginine	Simulated Drug Product	Validation	30	From (Massei et al. 2023)
SA1	6% Sucrose + 1% Arginine	Simulated Drug Product	Validation	28	From (Massei et al. 2023)
SA3	6% Sucrose + 3% Arginine	Simulated Drug Product	Validation	31	From (Massei et al. 2023)
S3	3% Sucrose	Placebo	Validation	63	From (Massei et al. 2023)
S6	6% Sucrose	Placebo	Validation	91	From (Massei et al. 2023)
T6	6% Trehalose	Placebo	Validation	45	From (Massei et al. 2023)

Product 1 and Product 2 were part of the *ad‐hoc* dataset. Being the largest placebo dataset available, Product 1 dataset was used for training and testing purposes, which are the most resource‐intensive steps of algorithm development. The second, composed of drug products, was used for validating the performances of models trained on placebo when dealing with unknown drug product samples. SA05, SA1, SA3 were Simulated Drug Products sourced from literature. Unlike Product 2, they could not be considered a standard Drug Product since they were sucrose‐based solutions containing a varying percentage of arginine. This amino acid was inserted to mimic the presence of the active principle without incurring the collateral costs and waste associated with the combined use of products containing active principle and disruptive techniques as the Karl Fischer Titration. Despite this, the data were included in the dataset to prevent the occurrence of overfitting models for Product 1 and to explore formulations with a wider range of amino acid concentrations.

S3, S9 and T6 were placebo sets engaged for evaluating model performances in predicting residual moisture in unknown placebo samples. The first two contain different sucrose percentages while the third contains a diverse type of sugar, trehalose. These specific features allow for individually investigating the impact on performance that placebo samples with different characteristics compared to the training dataset might have. The spectra of placebo and Drug Products in the whole range of wavelength investigated are reported in Figure S1 (Section 2 of Supporting Information).

### 2.5 Machine Learning Models Development

The dataset was analyzed and modeled using different regression approaches: Partial Least Squares (PLS) regression, Support Vector Regression (SVR), XGBoost, and an ensemble model combining both PLS and SVR. Each model was chosen for its specific advantages in handling high‐dimensional, complex datasets and addressing the challenges of residual moisture prediction. Detailed information on the model development strategies, hyperparameter tuning, and algorithm‐specific configurations for the Machine Learning models used are reported in Section 3 of Supporting Information.

#### 2.5.1 Partial Least Squares (PLS)

PLS is a supervised statistical‐based method modelling the relationships between the independent (X) and dependent (Y) variables by projecting both into a new latent space defined by a set of score vectors. In this case, latent variables are meant to maximize the covariance between X and Y, thus providing effective dimensionality reduction while preserving the most predictive features of the dataset (R. Rosipal and N. Krämer 2006, “Overview and recent advances in Partial Least Squares,” ed.). The PLS algorithm can be described through Equation 5:

(5) $$X=TP^{T}+E$$

where *T* is the score matrix, *E* is the residual, and *P* is the loading one. This technique proves particularly advantageous in the presence of a high‐dimensional dataset characterized by variables linearly correlated to each other. However, it also presents some drawbacks such as the strong dependence between the accuracy of the model and the number of components, and the risk of overfitting when a high number of components is incorporated. Further details on PLS development are included in Supporting Information (Section S3.2).

#### 2.5.2 Support Vector Regression (SVR)

The SVR is designed for non‐linear and complex datasets. Due to the kernel trick, they can be mapped into a multi‐dimensional space, where simpler correlations might be more easily identifiable. It is an extension of the SVM adopted to solve regression problems. The primary objective of SVM is to find a hyperplane capable of performing the separation of the space within the classes, whose direction and position are defined by the vectors. In other words, the role of these vectors is to define a decision boundary. They are points from the dataset chosen to maximize the distance within the vectors and the hyperplane, thereby improving the number of points correctly elaborated (Cervantes et al. 2020, “A comprehensive survey on support vector machine classification: Applications, challenges and trends,” ed.). Further details on SVR development are included in Supporting Information (Section S3.3).

#### 2.5.3 XGBoost

XGBoost (Extreme Gradient Boosting) is a scalable tree boosting algorithm that builds an ensemble of decision trees sequentially, where each new tree is trained to correct the residual errors left by the previous ones (Chen and Guestrin 2016). Such approach follows a gradient boosting framework in which the model is optimized by minimizing a regularized objective function incorporating both a loss term and explicit penalties on tree complexity. This regularization, combined with techniques such as column subsampling and shrinkage, makes XGBoost particularly effective at controlling overfitting while maintaining high predictive accuracy. The algorithm also handles sparse data natively and is computationally efficient thanks to its cache‐aware learning strategy. A known limitation is the relatively large number of hyperparameters to tune — including learning rate, maximum tree depth, and subsampling ratios — which can make model selection non‐trivial. Further details on XGBoost development are included in Supporting Information (Section S3.4).

#### 2.5.4 The Ensemble Model

In the end, the ensemble model is an algorithm derived from combining multiple regressors to obtain a single output. The outcome is generated by multiplying the predicted values obtained from the single models involved with specific weights and ultimately summing them. Being computationally intensive, it represents an alternative solution when a single model does not meet the predictive accuracy requirements or when determining the best model among those tested is challenging since their performances are similar. Further details on the ensemble model development are included in Supporting Information (Section S3.5).

#### 2.5.5 Models Implementation

The integration was carried out using Python (ver. 3.9) within the web‐based computational environment Jupyter Notebook. The PLS and SVR regression models were implemented using the scikit‐learn (ver. 1.2.2) (Pedregosa 2011) library, while XGBoost was implemented using the XGBoost Python package. The ensemble algorithm was implemented using a combination of scikit‐learn and custom ensemble method written in Python.

Prior to model development, all spectra were preprocessed using a Savitzky‐Golay filter (window_size = 31, second‐order polynomial), followed by Standard Normal Variate (SNV) normalization. The internal dataset was then randomly divided into training and test subsets using a 70‐30 split (with random state = 0), while independent external datasets, sourced both in the present study and from the literature, were reserved exclusively for final validation.

All the hyper parameters were selected performing grid search. PLS was developed using a number of 10 latent variables, selected according to the optimized conditions reported in the Supporting Information, while the hyper parameters of SVR and XGBoost were optimized coupling such approach with five‐fold cross‐validation. For SVR, the optimization included the regularization parameter (C), the epsilon‐insensitive loss parameters (ε) and the kernel function (linear, radial basis function and polynomial). For XGBoost, the number of estimators, maximum tree depth and learning rate were optimized. More information on the hyperparameters selection is contained in the Supporting Information (Section S3).

To further assess model robustness, all final models were additionally evaluated by independent five‐fold cross‐validation, and the mean and standard deviation of RMSE and R^2^ score were calculated.

The models introduced were first tested internally by evaluating the accuracy of the predictions when dealing with known data and then externally, by employing unknown data from literature. The parameters evaluated in these two phases are (James et al. 2021):

− Coefficient of determination (R^2^ score, Equation 6 (James et al. 2021)) on testing, training, and external validation, which provides a normalized measure of the proportion of variance explained by the model, by ratioing the residual sum of squares (representing the unexplained variance by the model) and the total sum of squares (representing the total variance in the target variable):

(6) $$R^{2}=1-\frac{\sum_{i=1}^{n}{\left(y_{i}-{\hat{y}}_{i}\right)}^{2}}{\sum_{i=1}^{n}{\left(y_{i}-\bar{y}\right)}^{2}}$$

Such R^2^ has been also evaluated on cross‐validation and external validation (Wold et al. 2001).

− Root mean squared error (RMSE, Equation 7 (James et al. 2021)) on training, testing, and external validation, that describes the square root of the average of the squared differences between the predicted (${\hat{y}}_{i}$) and the actual ($y_{i}$) values:

(7) RMSE=1n∑i=1n(yi−y^i)2

− Absolute error (AE_i_, Equation 8 (James et al. 2021)) and mean absolute error (MAE, Equation 9 (James et al. 2021)) on training, testing, and external validation, that describe the absolute difference between the predicted value (${\hat{y}}_{i}$) and the actual value ($y_{i}$) and the average of all absolute errors across a dataset, respectively. Both AE and MAE are in the same units as the target variable. AE provides a simple measure of the error magnitude for single prediction, while MAE is a tool to compare the accuracy between models.

(8) AEi=|yi−y^i|

(9) MAE=1n∑i=1n|yi−y^i|

− Normalized root mean squared error (NRMSE, Equation 10) on training, testing and external validation, which is obtained by normalizing the RMSE by a reference value of the target variable, in this case corresponding to the difference between the maximum and minimum observed values, being a dimensionless index to compare predictive accuracy across datasets or models operating on different scales (Botchkarev 2019).

(10) $$\mathrm{NRMSE}=\frac{\mathrm{RMSE}}{y_{\max}-y_{\min}}$$

− Residual Predictive Deviation (RPD, Equation 11) on testing and external validation, defined as the ratio of the standard deviation of the reference values to the RMSE of prediction. RPD provides a scale‐independent measure of model performance, and it is particularly employed in spectroscopic applications to assess whether a model is suitable for screening (higher than 2) or is considered unreliable for predictive purposes (lower than 1.5) (Chang et al. 2001).

(11) $$\mathrm{RPD}=\frac{\mathrm{SD}}{\mathrm{RMSE}}=\frac{\sqrt{\frac{1}{n-1}\sum_{i=1}^{n}{\left(y_{i}-\bar{y}\right)}^{2}}}{\mathrm{RMSE}}$$

#### 2.5.6 Generalization Performances of the Ensemble Model

When the relationship between dependent and independent variables does not follow a unique trend, two or more models may exhibit higher accuracies when processing specific range of data. Developing an ensemble model could aid in addressing complex distribution, as it possesses the capability to unify the strength of multiple models. In order to combine them, defining one or more points of transition may be necessary. Those are independent variable setpoints that represent a rigid boundary for the algorithm during calibration. In this phase, the algorithm determines the best model to use for each prediction according to the position of the sample with respect to this settled threshold.

This strategy consents to increasing the performance of the model in the absence of a specific data trend but it also introduces an additional risk of overfitting it since the placement of these boundary values may be specific to the training dataset. A robustness test may verify whether the ensemble model can deal with unknown sets of data with sufficient accuracy. The data utilized are sourced from the research conducted by Massei et al. 2023 (Massei et al. 2023). The samples analyzed are placebo samples plus arginine (mimicking different concentrated drug products) and placebo, formulated as follows:

− 6% sucrose‐based solution with 0.5% arginine (SA05, 30 samples) and 1% arginine (SA1, 28 samples).
− 3% sucrose‐based solution with 3% arginine (SA3, 31 samples).
− 6% trehalose‐based solution (T6, 45 samples).
− 3% sucrose‐based solution (S3, 63 samples).
− 9% sucrose‐based solution (S9, 36 samples).

It is important to note that the drug product formulations exhibit API concentrations approximately four to five orders of magnitude higher than those present in the drug product used in this study, making the dataset highly challenging for the ensemble model.

## 3 Results and Discussion

### 3.1 Performances of PLS, SVR, XGBoost, and Ensemble Models on the Ad‐Hoc Dataset

In Massei et al. work (Massei et al. 2023), models with mean absolute error lower than 0.5% were considered industrially promising as an alternative to Karl Fischer titration. The performance metrics for training, testing, and validation of the three models, evaluated using the ad‐hoc dataset, are presented in Figure 1 and Table 7.

**Figure 1:**
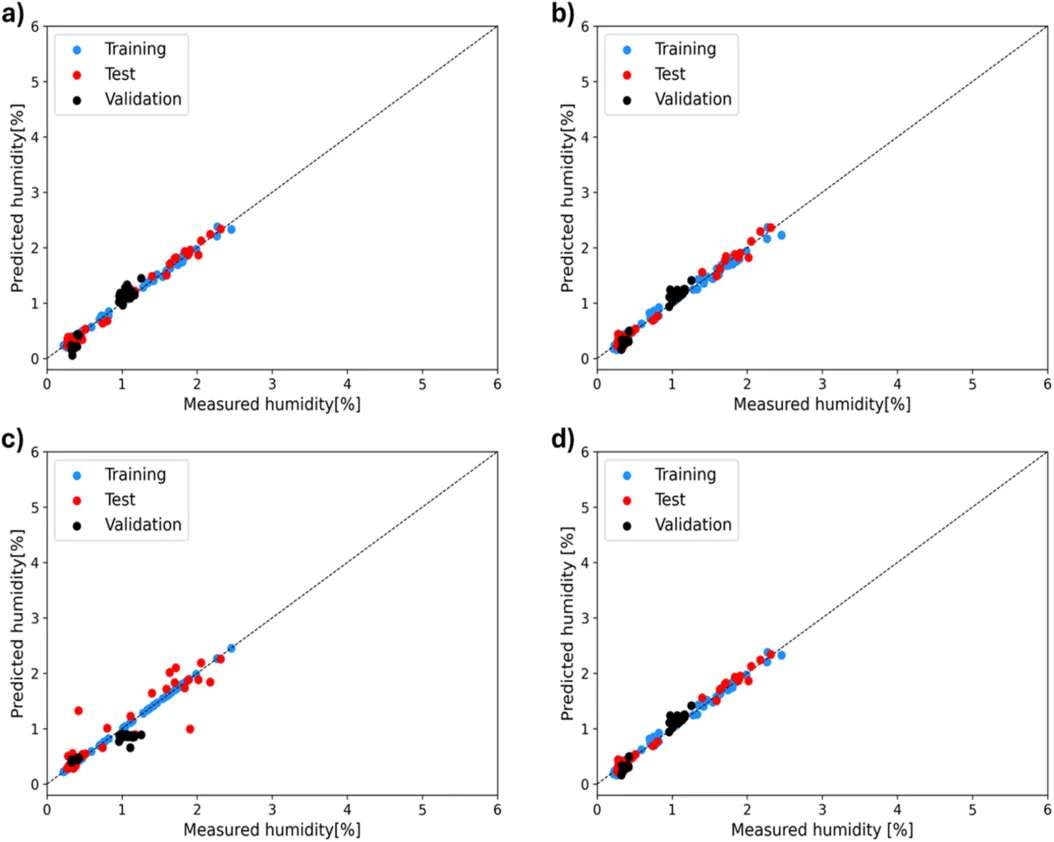
Predicted humidity versus measured humidity for PLS model (a), SVR model (b), XGBoost model (c), and ensemble model (d) on the ad‐hoc dataset.

**Table 7:** Performance metrics for each phase of the models with the ad‐hoc dataset.

Model	R^2^ training	R^2^ test	R^2^ validation	RMSE training	RMSE test	RMSE validation	NRMSE validation
PLS	0.996	0.987	0.800	0.037%	0.077%	0.146%	0.157
SVR	0.988	0.986	0.902	0.068%	0.081%	0.102%	0.110
XGBoost	0.999	0.867	0.620	0.001%	0.254%	0.201%	0.217
Ensemble	0.991	0.987	0.902	0.058%	0.079%	0.101%	0.110

PLS, SVR and XGBoost had similar values of R^2^ during training and testing, denoting that all the models can be successfully applied to assess Residual Moisture in known placebo samples.

About the PLS and SVR, the R^2^ score related to testing and training remains between 0.988 and 0.996 while for XGBoost such value is significantly lower (0.867).

However, the difference between the performance parameters of the SVR and the PLS widens when the placebo‐based trained algorithms make predictions on drug product samples. Looking solely at the parameter R^2^ values listed in Table 7, SVR performances appear significantly higher than that of the PLS during external validation having an R^2^ value of 0.902 instead of 0.800, whereas XGBoost performance remains notably poor with a R^2^ value of 0.620, clearly indicating the presence of strong overfitting. Such consideration is confirmed by the drop in the ratio of performance to deviations (RPD, included in Table 8) values both in test and validation stages.

**Table 8:** Cross‐validation performance metrics and Ratio of Performance to Deviation (RPD) of the models with the ad‐hoc dataset.

Model	CV R^2^ (mean ± SD)	CV RMSE mean	CV RMSE SD	RPD training	RPD test	RPD validation
PLS	0.981 ± 0.006	0.086%	0.010%	17.18	9.16	2.28
SVR	0.978 ± 0.009	0.090%	0.021%	9.43	8.65	3.27
XGBoost	0.881 ± 0.086	0.201%	0.065%	1209.71	2.78	1.65
Ensemble	0.983 ± 0.005	0.083%	0.006%	11.11	8.94	3.26

Furthermore, combining the information from Figure 1 and Table 7, it can be noted that the gap within PLS predicted and measured values during training, testing, and validation decreases progressively at higher level of humidity, making PLS emerge as more efficient than SVR in training and testing phases for moisture levels above ~1.4%. For this reason, such value was then empirically selected as the model‐switching threshold for the ensemble model. Disparities observed within the parameters contained in Table 7 and the plot in Figure 1 are reasonable as Product 2 dataset contains a non‐uniform residual moisture distribution with an upper moisture limit of 1.4%. Indeed, the algorithms are validated using drug products falling in a moisture range below the one where PLS appears to surpass SVR in training and testing performances. Hence, the creation of an ensemble model and its further generalization is necessary to extend the range of humidity where the algorithm can make accurate predictions on placebo and to assess overall performances in presence of unknown samples. As expected, metrics reported in Table 7 confirm how the ensemble model combines the strength of PLS and SVR.

SVR and ensemble models essentially achieve identical validation accuracy. However, by analyzing the cross‐validation performance included in Table 8, the ensemble model clearly distinguishes itself (CV RMSE SD = 0.006% *vs.* 0.021% for SVR) through cross‐validation stability, indicating more consistent generalization across data partitions and making the ensemble model the most robust and reproducible choice overall.

The distribution of absolute errors (%) relative to the predictions of RM of PLS, SVR, XGBoost, and ensemble models, depicted in Figure 2, further confirm the effectiveness of this approach.

**Figure 2:**
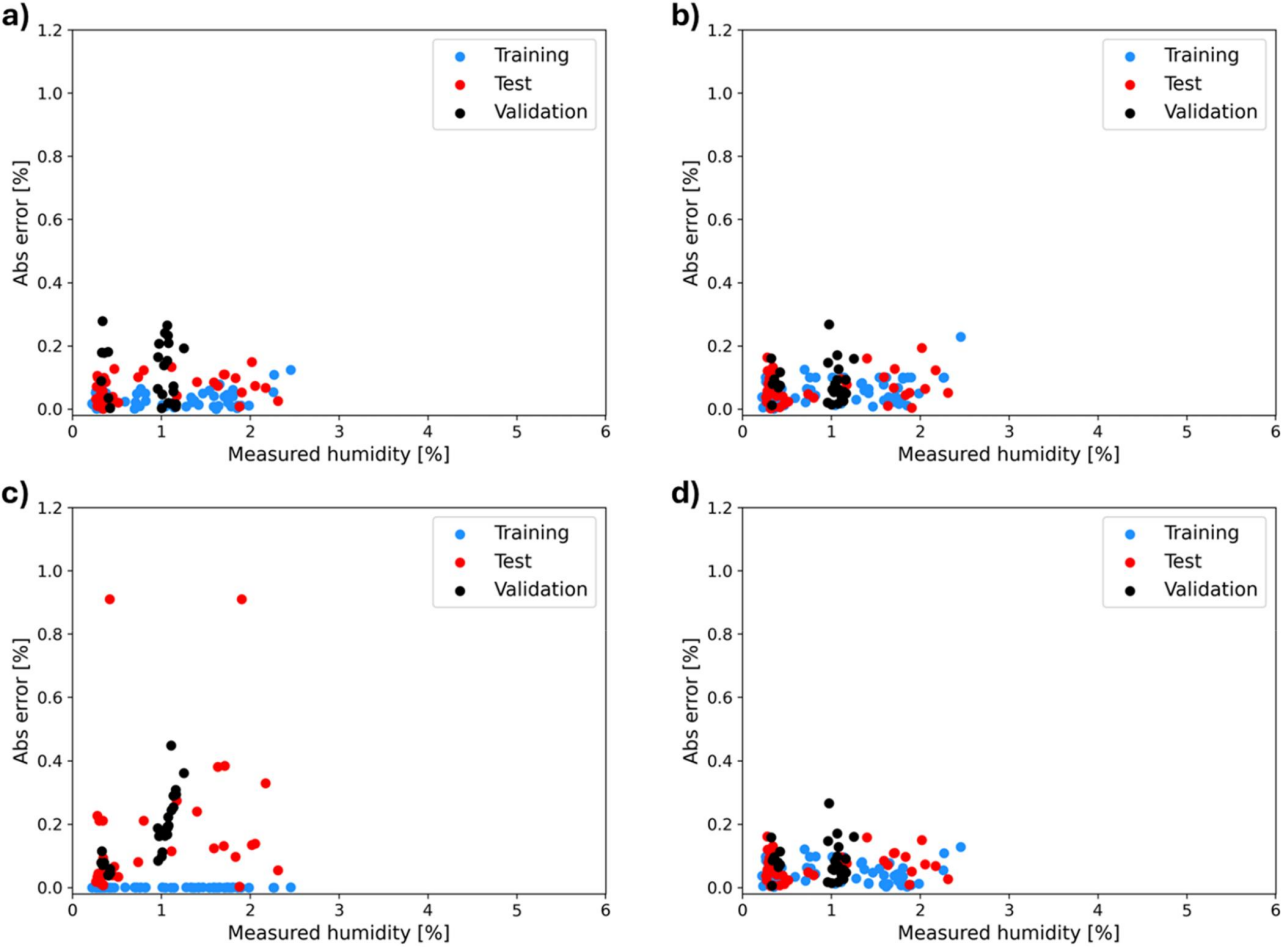
Absolute error [%] of PLS model (a), SVR model (b), XGBoost model (c), and ensemble model (d) on the ad‐hoc dataset.

The absolute error plots confirm all the observations anticipated. PLS is more accurate during training and testing phases, especially at higher moisture levels. Its presence in high moisture range decreases the RMSE for the ensemble model, both in training and testing, from 0.068% and 0.081% (for the SVR) to 0.058% and 0.079%, respectively.

Regarding the validation, samples from the *ad‐hoc* dataset do not provide an objective point of view on the applicability of PLS, as the moisture range of the data used is limited to the values where PLS performs poorly. However, by looking at the distribution of the error versus the measured humidity, we can state that PLS is less adaptive when dealing with unknown samples containing lower moisture level, confirming that replacing PLS with SVR in such cases can extend the ensemble model's applicability to non‐placebo products.

### 3.2 Adaptability of the Ensemble Model to Specific Drug Product Samples

In the pharmaceutical field, stability studies are generally conducted on specific targets, for which an exceptionally high level of accuracy in predicting the required CQA is strongly desired. Therefore, tailoring the ensemble model to predict Residual Moisture for a specific type of drug product can enhance its usefulness in industrial applications. The easiest approach is to enrich the training dataset by adding a small number of such samples, slightly impacting on its development costs and the experimental campaign needed.

Figure 3 shows the performance obtained by training the ensemble model on a dataset composed of 134 samples, where 129 are placebo and 5 are drug products, both taken from the ad‐hoc dataset.

**Figure 3:**
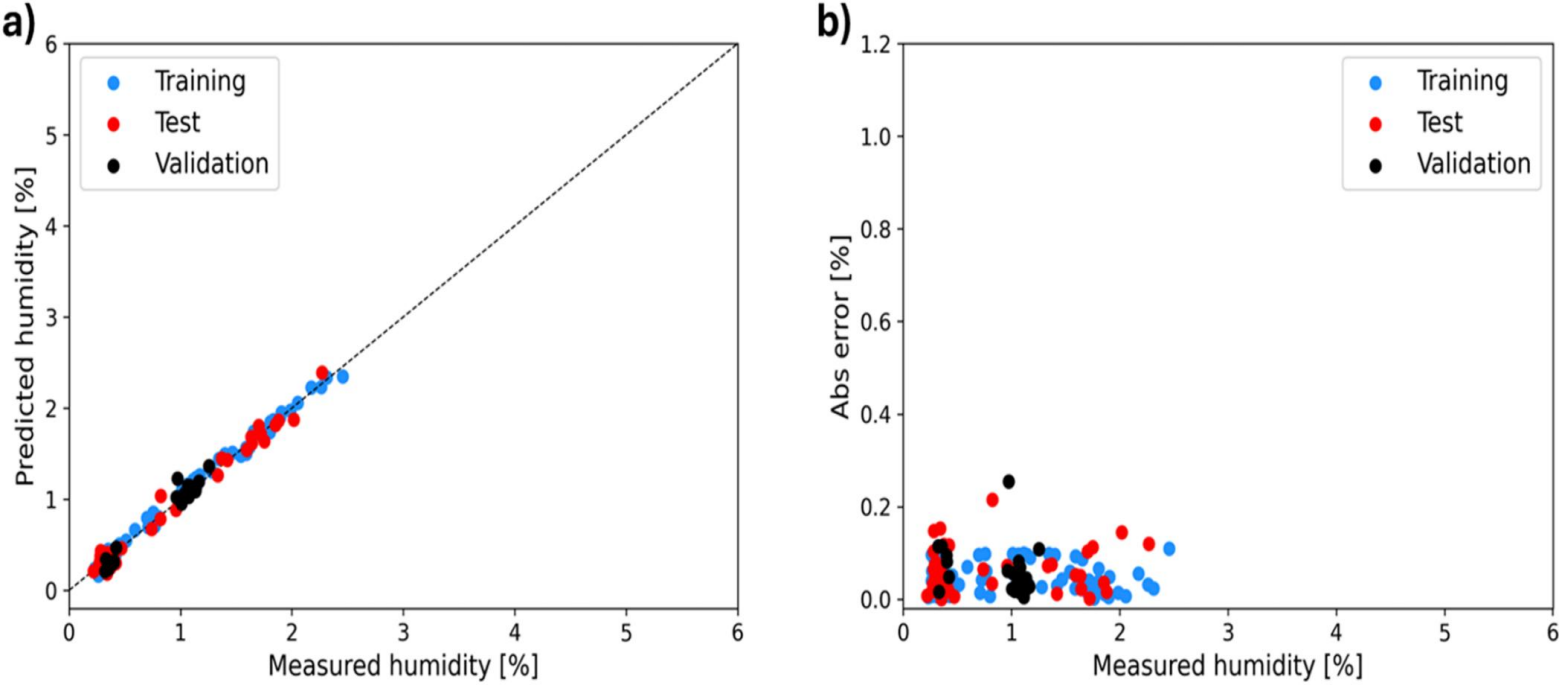
Predicted humidity versus measured humidity (a) and absolute error [%] (b) of the ensemble model particularized for samples with DPs (Product 2 set), validated on DPs (Product 2 set).

The results obtained (Table 9) are promising since the validation R^2^ score increases from 0.902 to 0.935 with no relevant impact on the training and testing performances, denoting the possibility to adapt the algorithm to a large variety of products with limited efforts.

**Table 9:** Predicted humidity versus measured humidity (a) and absolute error [%] (b) of the ensemble model particularized for samples with DPs (Product 2 set), validated on DPs (Product 2 set).

Model	R^2^ training	R^2^ test	R^2^ validation	RMSE training	RMSE test	RMSE validation	NRMSE validation
Ensemble	0.991	0.984	0.935	0.061%	0.080%	0.082%	0.088

Overall, Table 9 confirms that a minimal, cost‐effective augmentation strategy – requiring as few as 5 DP samples alongside the placebo training set – drives meaningful performance improvements.

### 3.3 Generalization of the Ensemble Model to External Datasets

The results obtained from the generalization (Figure 4) confirmed that the ensemble algorithm trained exclusively on placebo samples can deal successfully with Residual Moisture prediction in unknown drug product samples (Figure 4a). Indeed, as shown in Table 10, the R^2^ validation score for the Mixed DP dataset reached 0.953, while the one for the SA3 dataset is 0.874. This indicates a moderate, but not critical, decline in predictive performance. Notably, the SA3 dataset was considered the most challenging test case in this study. This slight reduction in performance could be considered reasonable, given that the model was trained on samples with lower Residual Moisture levels than those found in the SA3 dataset.

**Figure 4:**
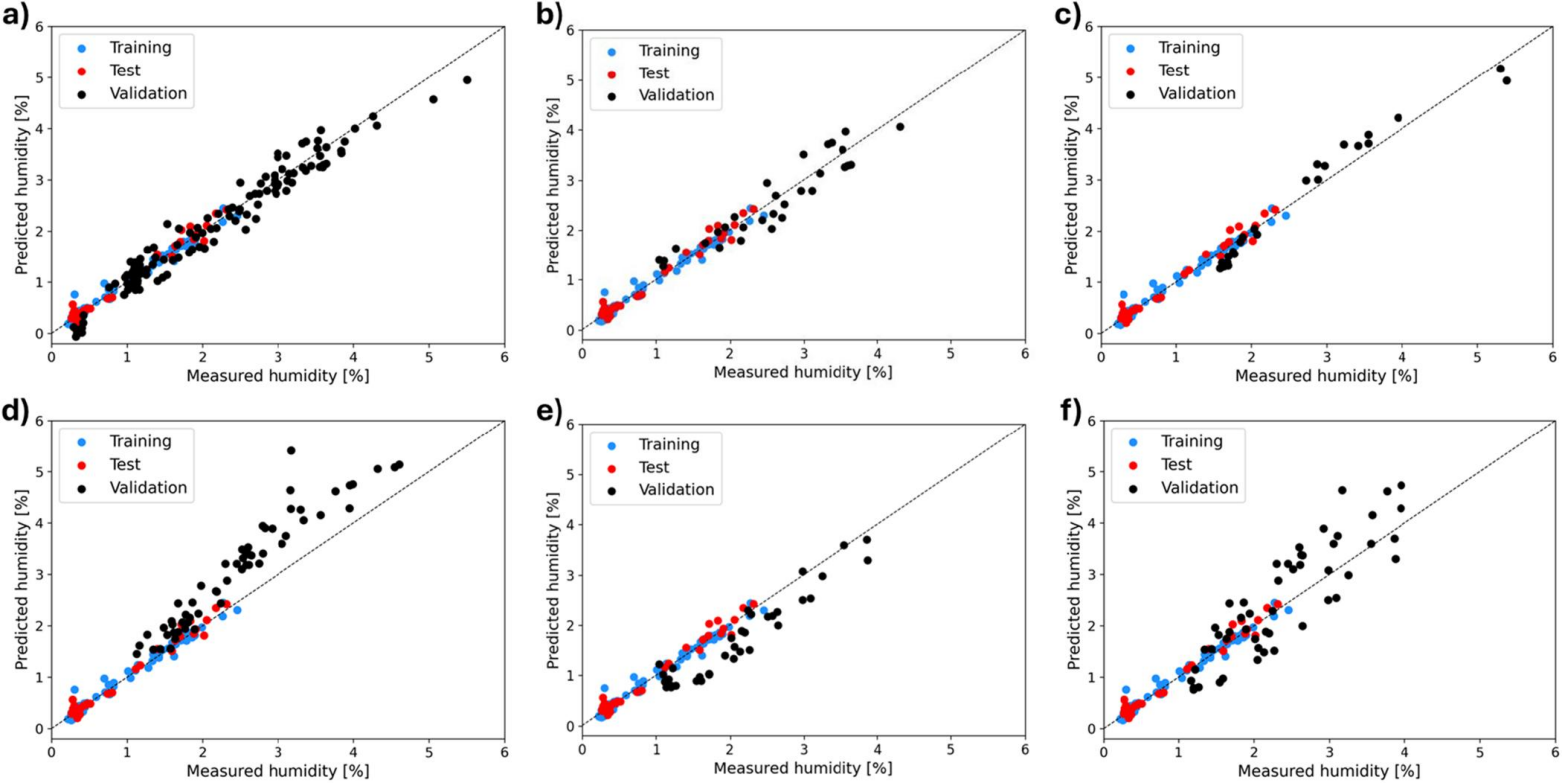
Predicted humidity versus measured humidity for the ensemble model on: (a) mix DP, (b) SA3, (c) T6, (d) S3, (e) S9, (f) S3 + S9; datasets from Massei et al. 2023 (Massei et al. 2023).

**Table 10:** Performance metrics for validation phase of the ensemble model with different literature‐sourced datasets (from (Massei et al. 2023)).

Dataset	R^2^ validation	RMSE validation	NRMSE validation	RPD validation
Mixed DPs	0.953	0.244%	0.047	4.65
SA3	0.874	0.301%	0.092	2.87
T6	0.940	0.273%	0.072	4.15
S3	0.343	0.709%	0.204	1.24
S9	0.679	0.453%	0.160	1.79
S3 + S9	0.506	0.571%	0.205	1.44

Another aspect to investigate is the impact of the sugar type and concentration of the samples in the training set on the model's performance. About the first case, the ensemble algorithm preserves its high performance as shown in Figure 5.

**Figure 5:**
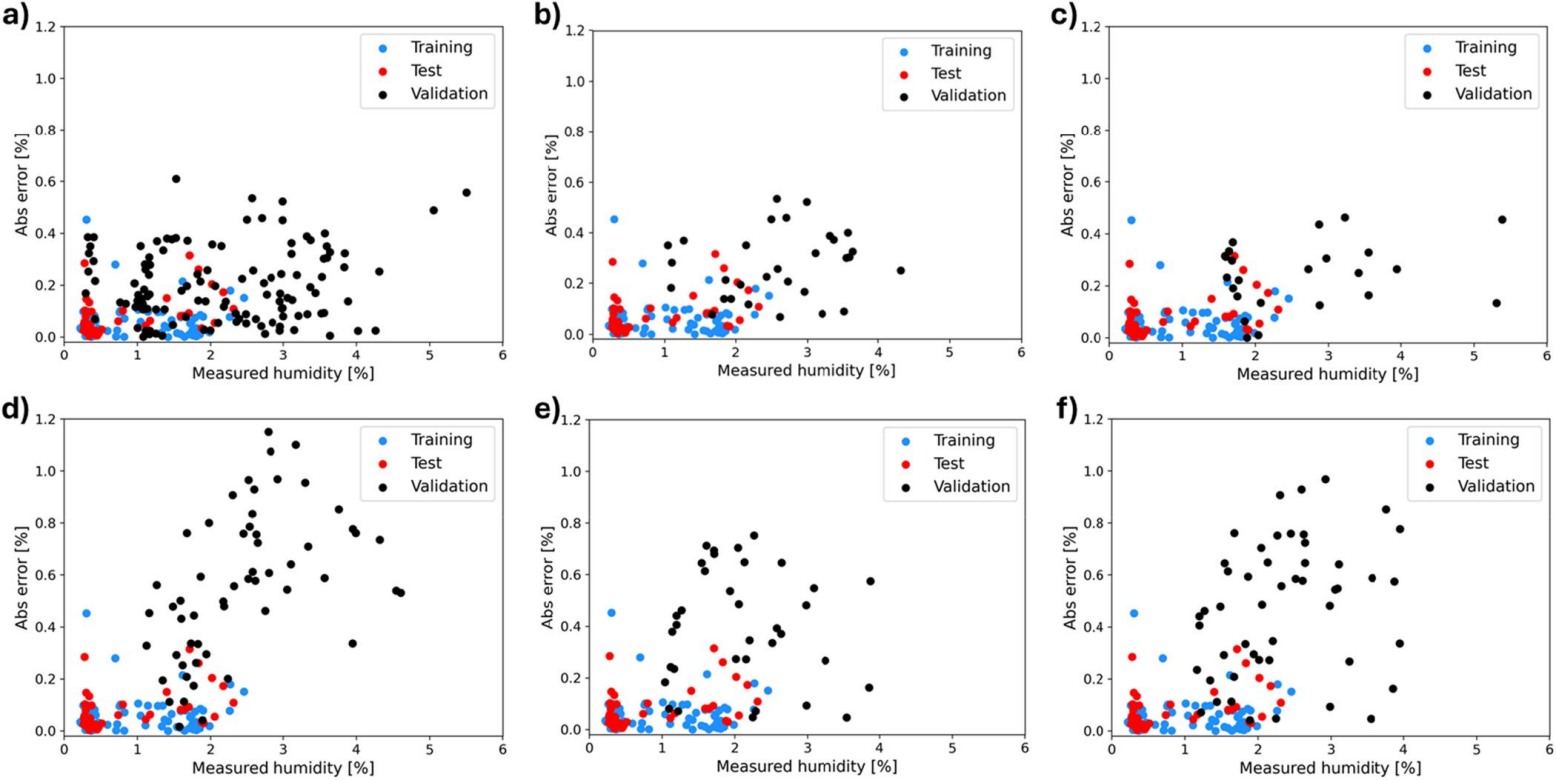
Absolute error [%] for the ensemble model on: (a) mix DP, (b) SA3, (c) T6, (d) S3, (e) S9, (f) S3 + S9 datasets from Massei et al. 2023 (Massei et al. 2023).

The presence of placebo samples having different sugar concentrations is one of the major challenges of the ensemble algorithm trained exclusively on placeboes containing a defined percentage of sugar. As shown in Table 10, the R^2^ score decreased respectively to 0.343 for the S3 samples and to 0.679 for the S9 samples. In these specific cases, the algorithm cannot be considered effective for Residual Moisture predictions.

### 3.4 Adaptability of the Ensemble Model to Different Composition Samples From External Datasets

As previously discussed, the ensemble PLS + SVR model can be adapted to specific cases by including a small number of representative samples in the training dataset. In this instance, placebo samples with varying sugar concentrations were prepared and included to enhance the model's adaptability. This strategy is both cost‐effective and practical for industrial applications, particularly when tailoring models for DPs. The modified training dataset was constructed as follows:

− The S9 sample set was divided into two subsets; one‐third (12 samples) were allocated for training (S9 training), while the remaining two‐thirds (24 samples) were reserved for testing (S9 testing);
− Similarly, the S3 sample set was split equally, with 32 samples used for training (S3 training), and 31 samples for testing (S3 testing).

The resulting mixed training dataset consisted of 129 samples in total: 32 samples from S3, 12 samples from S9, 85 placebo samples from the current study. This configuration was designed to address the previously identified challenges in predictions. Earlier results indicated that the ensemble model struggled more in predicting S3 dataset compared to S9. Therefore, increasing the representation of S3 samples in the training set aimed to improve the model's ability to manage these more complex samples effectively.

As anticipated, the inclusion of samples with specific concentrations of excipients into the training dataset led to a significant improvement in validation performance (Figure 6).

**Figure 6:**
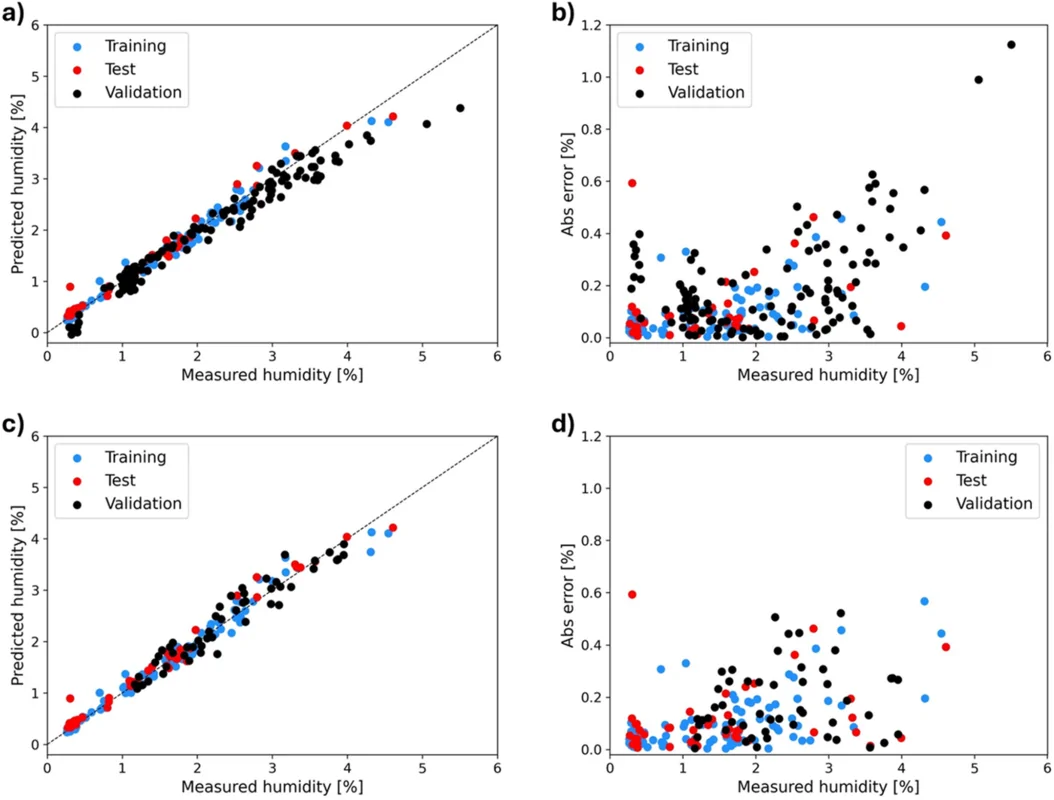
Predicted humidity versus measured humidity of the ensemble model particularized for samples with different excipients, validated on mixed DPs (a) and on S3 + S9 samples (c). Absolute error [%] of the ensemble model particularized for samples with different excipients, validated on mixed DPs (b) and on S3 + S9 samples (d). Dataset from Massei et al. 2023 (Massei et al. 2023).

The enhanced ensemble model showcased an improved ability to predict residual moisture in samples with varying sugar concentrations. For instance, the validation R^2^ score for the S3 + S9 dataset increased from 0.506 to 0.922, with a reduced validation RMSE of 0.226% (Table 11).

**Table 11:** Performance metrics for each phase of the ensemble model particularized with specific datapoints sourced from literature (from (Massei et al. 2023)). Validated on datasets from Massei et al. 2023 (Massei et al. 2023).

Dataset	R^2^ training	R^2^ test	R^2^ validation	RMSE training	RMSE test	RMSE validation	NRMSE validation
Mixed DPs	0.978	0.976	0.940	0.145%	0.172%	0.274%	0.053
S3 + S9	0.978	0.976	0.922	0.145%	0.172%	0.226%	0.081

Additionally, the adjustment of the training dataset maintained robust predictive performances for mixed drug product samples (Figure 6a,b), achieving a validation R^2^ of 0.940 and a validation RMSE of 0.274% (Table 11). This reflects a substantial improvement in the algorithm's applicability while reducing the number of experimental points to be included. This adaptation of the ensemble model demonstrates its effectiveness in handling samples with different sugar concentrations while maintaining strong predictive accuracy for DPs. These results highlight the model's flexibility and suitability for broader applications, further supporting its industrial relevance.

## 4 Conclusions

The application of PAT (i.e., Process Analytical Technology) in biopharmaceuticals, particularly for real‐time monitoring and control, is a critical step toward ensuring product quality and process efficiency. Other cutting‐edge technologies such as artificial intelligence and machine learning showcase great opportunities to evaluate, predict, and extract desired insights either through understanding the patterns in the data or by establishing mathematical relationships between CPPs (*i.e*., Critical Process Parameters) and CQAs (*i.e*., Critical Quality Attributes) of biopharmaceutical products (Puranik et al. 2022). In this light, a major contribution of this work was the development of a PAT tool combining NIR (*i.e*., Near‐Infrared) spectroscopy and machine learning modelling for at‐line residual moisture evaluation in lyophilized products, which is a pivotal CQA for this product category, which directly impacts stability, efficacy, safety, and shelf‐life. Reliable and accurate RM (*i.e*., Residual Moisture) evaluation is essential for ensuring product quality during its manufacturing and storage. Traditional well‐established methods, such as Karl‐Fischer titration, are destructive, time‐consuming, and resource‐intensive. For these reasons, only part of the vials within a batch can be analyzed through this technique, leading to a poor process understanding. Indeed, the integration of PAT principles offers a great opportunity to design, develop, and implement advanced tools for real‐time quality monitoring, streamlining pharmaceutical processes, and improving overall efficiency. In this context, the ensemble machine learning model presented in this study has proven to be a robust and adaptable solution for RM evaluation from NIR spectra of lyophilized biopharmaceuticals. The model combines PLS (*i.e*., Partial Least Squares) and SVR (*i.e*., Support Vector Regression) methods, leveraging their respective strengths to deliver high‐predictive performance within different ranges of residual moisture with minimal compromise in terms of models explainability. This underscores the complex, non‐linear relationships between RM values and spectral features. It has showcased sufficient accuracy and low RMSE (*i.e*., Root Mean Squared Error) in predicting RM for both placebo and DP (*i.e*., Drug Product) samples, making it a promising PAT tool for both fundamental research and industrial applications. The novelty of this work lies in its ability to leverage a limited dataset of placebo samples to predict residual moisture in more complex DPs, including those with significantly higher API content. Such generalization is rarely addressed in prior NIR‐ML studies. Moreover, the ensemble model's adaptability, evidenced by performance improvements following the inclusion of a small number of target‐specific samples, further enhances its practicality by reducing development costs and timelines.

The PLS‐SVR ensemble model has emerged as the most robust and reproducible solution: it achieved competitive validation accuracy on DP samples while exhibiting the lowest cross‐validation RMSE standard deviation across folds, reflecting superior stability compared to SVR, PLS, and XGBoost individually.

Despite these promising results, additional investigations are needed to fully unlock the model's potential. While the 1.4% model‐switching threshold is empirical, it reflects underlying spectral behaviour: high‐moisture samples exhibit broader, more linear NIR signals, thus favoring PLS, while low‐moisture samples require the nonlinear flexibility of SVR. Further analyses may unravel the physicochemical basis of this transition, potentially enabling more informed threshold selection or dynamic switching strategies. In addition, previous studies including those by Massei et al (Massei et al. 2023)., have identified challenges associated with highly concentrated APIs, reducing predictive accuracy. Encouragingly, the ensemble model here developed performed well on SA3 samples, being the DP with the highest API concentration and hence the worst‐case scenario; however, further work is required to determine the maximum API concentrations the model can handle without decreasing its accuracy.

Overall, the ensemble model represents a significant step forward in the application of machine learning to RM prediction in lyophilized biologics. By enabling rapid, non‐destructive, and accurate analysis, the developed tool meets the demands of PAT frameworks, thus potentially accelerating the transition of biopharmaceutical products from research to industrial stages. Furthermore, the proposed strategy potentially aligns with regulatory expectations for PAT as outlined in ICH Q13 and ICH Q14, supporting robust and explainable data‐driven control strategies during DP lyophilization process.

## Author Contributions

**Samuele Fiorenza:** methodology, software, validation, formal analysis, investigation, data curation, writing – original draft, writing – review and editing, visualization. **Mariapia D'Urso:** methodology, software, validation, formal analysis, investigation, data curation, writing – original draft, writing – review and editing, visualization. **Ambra Massei:** methodology, validation, formal analysis, investigation, writing – review and editing. **Nunzia Falco:** conceptualization, methodology, software, validation, formal analysis, investigation, data curation, writing – original draft, writing – review and editing, visualization. **Davide Fissore:** conceptualization, methodology, software, validation, formal analysis, investigation, data curation, writing – original draft, writing – review and editing, visualization. **Enza Torino:** conceptualization, methodology, software, validation, formal analysis, investigation, data curation, writing – original draft, writing – review and editing, visualization.

## Conflicts of Interest

The authors declare no conflicts of interest.

## Supporting information


Supporting File

## Data Availability

The data that supports the findings of this study are available in the supporting information of this article. The data that support the findings of this study, as well as the codings, are available from the corresponding author upon reasonable request.
