# Supplementary material for: Machine Learning‐Based PAT Tool for Real‐Time Residual Moisture Monitoring in Lyophilized Biopharmaceuticals Using NIR Spectroscopy
Source: Biotechnol Bioeng. 2026 Jul 12;123(11):3030–46. doi: 10.1002/bit.70302 (PMC13665612; doi:10.1002/bit.70302)
Supplement: Supplementary file 1 [file BIT-123-3030-s001.docx]

**SUPPORTING INFORMATION**

**S1. Lyophilization Process Parameters**

All Product 1 samples (*i.e.,* placebo) were freeze-dried using a pilot-scale lyophilizer (IMA Life, Italy) following a multi-step cycle designed to ensure high cake quality and reproducible Residual Moisture (RM) profiles. The lyophilization process was optimized in a different study, based on prior development runs and literature guidelines. The complete lyophilization cycle is detailed in table S1.

| **Step** | **Target Temperature** | **Duration** | **Ramp Time** | **Chamber Pressure** |
| --- | --- | --- | --- | --- |
| Freezing (step 1) | -45°C | 2 h | 1 h 30 m | Atmospheric |
| Annealing | -15°C | 1 h | 1 h | Atmospheric |
| Freezing (step 2) | -45°C | 4 h | 1 h | Atmospheric |
| Primary Drying | -20°C | 26 h | 50 m | 0.05 mBar |
| Secondary Drying | +35°C | 13 h | 9 h | 0.05 mBar |

***Table S1*** *– Lyophilization cycle, process parameters.*

**S2. NIR spectra**

As a first outcome, the spectra of placebo and Drug Products were comparable, since the profiles match in terms of shape in the whole range of wavelength investigated (Figure S1).

**Figure S1** - Preprocessed placebo (a) and drug product (b) NIR spectra.

Similarly, spectra recorded with different orientations of the vials had no significant differences (Figure S2), especially in the water peak region (5100 cm-1) (Figure S3), as confirmed by previous work [1]. Therefore, all the information inserted in the dataset only derived from the vertical set.

**Figure S2** - Horizontal (a) and vertical (b) oriented spectra for Placebo and their comparison (c).

**Figure S3** - Horizontal (a) and vertical (b) oriented spectra for Placebo and their comparison in the water peak range.

The preceding results illustrate that the exclusive use of placebo samples as training data does not represent a limitation for the industrial applicability of the models presented.

**S3. Model Development Details**

To support the main manuscript, this section provides detailed information on the model development strategies, hyperparameter tuning, and algorithm-specific configurations for the three Machine Learning approached used: Partial Least Squares (PLS), Support Vector Regression (SVR), XGBoost and the ensemble model.

**S3.1 Software Environment**

- Programming Language: Python
- Development Environment: Jupyter Notebook
- Libraries:
  - scikit-learn [2] (1.2.2) for regression models and grid search
  - XGBoost [3] (3.3.0.0)
  - numpy [4], pandas [5], matplotlib [6] for data processing and visualization

**S3.2 Partial Least Squares (PLS)**

The PLS model was developed to capture linear correlations between preprocessed NIR spectra and Residual Moisture (RM) values. Key features of the model construction include:

- Number of Latent Variables: 10 components; this number was selected via GridSearchCV, employing the mean square error on prediction as loss function
- Validation Strategy: 5-fold cross-validation
- Library Used: PLSRegression class from scikit-learn [2]

The model provided optimal performance in the higher moisture range (above ~ 1.4%).

**S3.3 Support Vector Regression (SVR)**

SVR was adopted to address nonlinearities in the relationship between spectral features and moisture content. Hyperparameter tuning was conducted via grid search (GridSearchCV) by using the mean square error on prediction as loss function, testing the following parameter combinations:

- Kernel: **Radial Basis Function (RBF)**, Polynomial (Poly), Linear
- Regularization Parameter C: [0.1, **1**, 10]
- Epsilon (ε-insensitive loss): [**0.1**, 0.2, 0.5]

Best performing model settings are reported in bold. The RBF kernel was selected for its capacity to model subtle nonlinear trends in spectral data. SVR outperformed PLS in the lower moisture range (<1.4%).

**S3.4 XGBoost**

Hyperparameter tuning in XGBoost was performed via GridSearchCV, testing the following parameter combinations:

Number of trees (n_estimators): [100, **200**, 300];

Maximum tree depth (max_depth): [2, **3**, 4, 6];

Learning rate: [0.03, 0.05, **0.1**];

Subsample: [**‘None’**, 0.8, 1.0];

Colsample_bytree: [**‘None’**, 0.8, 1.0];

L1 regularization (reg_alpha): **[‘None’,** 0.1];

L2 regularization (reg_lambda): [**‘None’**, 1.0, 5.0];

Minimum child weight: [**‘None’**, 1, 3].

The optimal hyperparameter combination (reported in bold) was selected by minimizing the mean squared error (MSE). Model performance was further assessed using 5-fold cross-validation, reporting both RMSE and R² metrics. XGBoost demonstrated competitive results across the entire moisture range exclusively in the presence of known samples.

**S3.5 Ensemble model**

The ensemble model combines predictions from the PLS and SVR models. A threshold strategy was applied to assign weights based on the predicted Residual Moisture range:

- Threshold: 1.4% RM
- For RM < 1.4%: SVR = 97%, PLS = 3%
- For RM > 1.4%: PLS = 97%, SVR = 3%

This approach allowed leveraging the strengths of both regressors across the RM spectrum. The ensemble model was implemented using custom Python code to combine output post-prediction.

**S 3.6 Model development pipeline**

In order to develop, optimize and validate the models introduced in this work, two datasets were employed: one used for model development and an independent external dataset reserved for validation.
For each workflow iteration, the internal dataset was partitioned into training an test subsets, while the external dataset was maintained as a hold-out validation set.

All models were developed following a three-step protocol, consisting of initial preprocessing optimization, model hyperparameter tuning and final training and evaluation.

**S.3.6.1 Preprocessing Optimization**

The spectral preprocessing was performed in the same ways for all the model by adopting the further preprocessing techniques:

- Second-Order Savitzky-Golay filter (window size = 31, polynomial order =2)
- Standard Normal Variate (SNV) transformation

Such data were finally collected into a dataset, from which the testing and the training dataset were sourced.

**S 3.6.2 Model Hyperparameter Optimization**

In the second step of the pipeline, model-specific hyperparameters were optimized using grid search. In all cases, model selection was based on maximizing the negative mean squared error. For PLS, the number of latent variables (*i.e.,* number of components) was optimized. For SVR, hyperparameters including the regularization parameter (C), the epsilon-insensitive loss, kernel type, and kernel coefficient were optimized via grid search. For XGBoost, hyperparameters including the number of boosting rounds (number of estimators, maximum tree depth, learning rate, subsampling ratio, column sampling ratio, and regularization parameters were optimized. The optimized hyperparameters values obtained are reported in bold in the relative section.

**S 3.6.3 Final training, testing and external validation**

For each model, final training was performed using optimized preprocessing- and hyper-parameters. Model performance was then evaluated on the training set, the internal test set and the external validation set. The following performance metrics were computed:

- R2 score for training
- R2 score for testing
- Q2 score for external validation
- NRMSE for training, testing and validation
- Root Mean Squared Error (RMSE) for all datasets
- RPD for training, testing and validation

**S 3.6.4 Cross-Validation for Statistical Robustness**

To ensure statistical reliability, K-fold cross-validation was performed on the training data for each final model. The mean and standard deviation of cross-validated performance metrics previously mentioned were reported. For the hybrid model, cross-validation was conducted by reconstructing both PLS and SVR models within each fold and applying the optimized combination strategy.

**Bibliography**

[1]	S. Bobba, N. Zinfollino, and D. Fissore, "Application of Near-Infrared Spectroscopy to statistical control in freeze-drying processes," vol. 168, ed. European Journal of Pharmaceutics and Biopharmaceutics, 2021, pp. 26-37.

[2]	F. a. V. G. l. a. G. A. a. M. V. a. T. B. a. G. O. a. B. M. a. P. P. a. W. R. a. D. V. a. Pedregosa, "Scikit-learn: Machine Learning in Python," *J. Mach. Learn. Res.,* vol. 12, no. null, pp. 2825–2830 , numpages = 6, nov 2011.

[3]	T. Chen *et al.*, "xgboost: Extreme Gradient Boosting," 2026 2026.

[4]	C. R. Harris *et al.*, "Array programming with NumPy," vol. 585, no. 7825, pp. 357-362, 2020.

[5]	W. McKinney, *Data Structures for Statistical Computing in Python*. 2010, pp. 56-61.

[6]	J. D. Hunter, "Matplotlib: A 2D Graphics Environment," *Computing in Science & Engineering,* vol. 9, no. 3, pp. 90-95, 2007, doi: 10.1109/MCSE.2007.55.
